# Endoscopic ultrasound-guided versus percutaneous transhepatic gallbladder drainage in surgically high-risk patients with acute cholecystitis: a systematic review and meta-analysis

**DOI:** 10.1007/s00464-026-13327-x

**Published:** 2026-09-22

**Authors:** Ricardo Antonio González-Jaramillo, Eduardo Jordan García, Andrea Melissa Briceño Gonzalez, Jorge Luis Rosales-Becerra, Brandon Duran-Suarez, Christopher Daniel Tadeo Hernández, Gabriela Alejandra Wong Ruiz, Carlos Javier Mata Quintero

**Affiliations:** 1 Department of General Surgery and Endoscopy, Hospital Central Norte, Petróleos Mexicanos (PEMEX), Mexico City, Mexico; 2 Department of Surgery, Hospital Ángeles Mocel, Mexico City, Mexico; 3 Faculty of Medicine, Universidad Nacional Autónoma de México (UNAM), Mexico City, Mexico

**Keywords:** Endoscopic ultrasound-guided gallbladder drainage, Lumen-apposing metal stents, Percutaneous transhepatic gallbladder drainage, Acute cholecystitis, Systematic review and meta-analysis, High surgical risk

## Abstract

**Background:**

Percutaneous transhepatic gallbladder drainage (PTGBD) has been the conventional non-operative option for acute cholecystitis in patients at high surgical risk, but endoscopic ultrasound-guided gallbladder drainage (EUS-GBD) with lumen-apposing metal stents (LAMS) is increasingly preferred because it removes the external catheter. We compared the two techniques, with device- or drainage-related unplanned readmission—the outcome specified in our public registration—as the primary endpoint.

**Methods:**

Four bibliographic databases and two trial registries were searched from inception to 31 January 2026, with the search updated to 30 July 2026. 6 comparative studies were included (one randomized trial and five observational cohorts; 712 patients, EUS-GBD 322 and PTGBD 390). Odds ratios (OR) were pooled with random-effects restricted maximum likelihood (REML) models using the Hartung–Knapp–Sidik–Jonkman adjustment, with leave-one-out and method-sensitivity analyses and GRADE assessment. The protocol was registered on the Open Science Framework before data extraction.

**Results:**

The primary outcome was reported at the patient level in three studies. Device- or drainage-related unplanned readmission occurred in 14/200 (7.0%) of EUS-GBD patients versus 91/245 (37.1%) of PTGBD patients. Every contributing study favoured EUS-GBD and each study-level interval excluded unity, but the pooled Hartung–Knapp interval did not (OR 0.098, 95% CI 0.008–1.22; *I*^2^ = 68%), and the estimate was not robust to leave-one-out omission. Technical success was lower with EUS-GBD in all six studies (technical failure 13/322 (4.0%) vs. 4/390 (1.0%); OR 0.31, 95% CI 0.087–1.11), although this interval also included unity. Clinical success (OR 0.99, 95% CI 0.33–2.99), 30-day adverse events (OR 0.55, 95% CI 0.024–12.57) and 30-day all-cause mortality (OR 0.97, 95% CI 0.076–12.51) did not differ significantly, with intervals too wide to exclude clinically important differences in either direction. Certainty was very low for the primary outcome and for adverse events, and low for the remainder.

**Conclusions:**

In surgically high-risk patients with acute cholecystitis, EUS-GBD achieved clinical success and mortality comparable to PTGBD, with a small and consistent numerical reduction in technical success, and with a device-related readmission burden that was lower in every contributing study but not robustly lower on pooled analysis. The present evidence does not establish EUS-GBD as a universally superior first-line replacement for PTGBD. Adequately powered randomized trials using standardized, patient-level endpoints and longer follow-up are required.

**Graphical abstract:**

**Figure:**
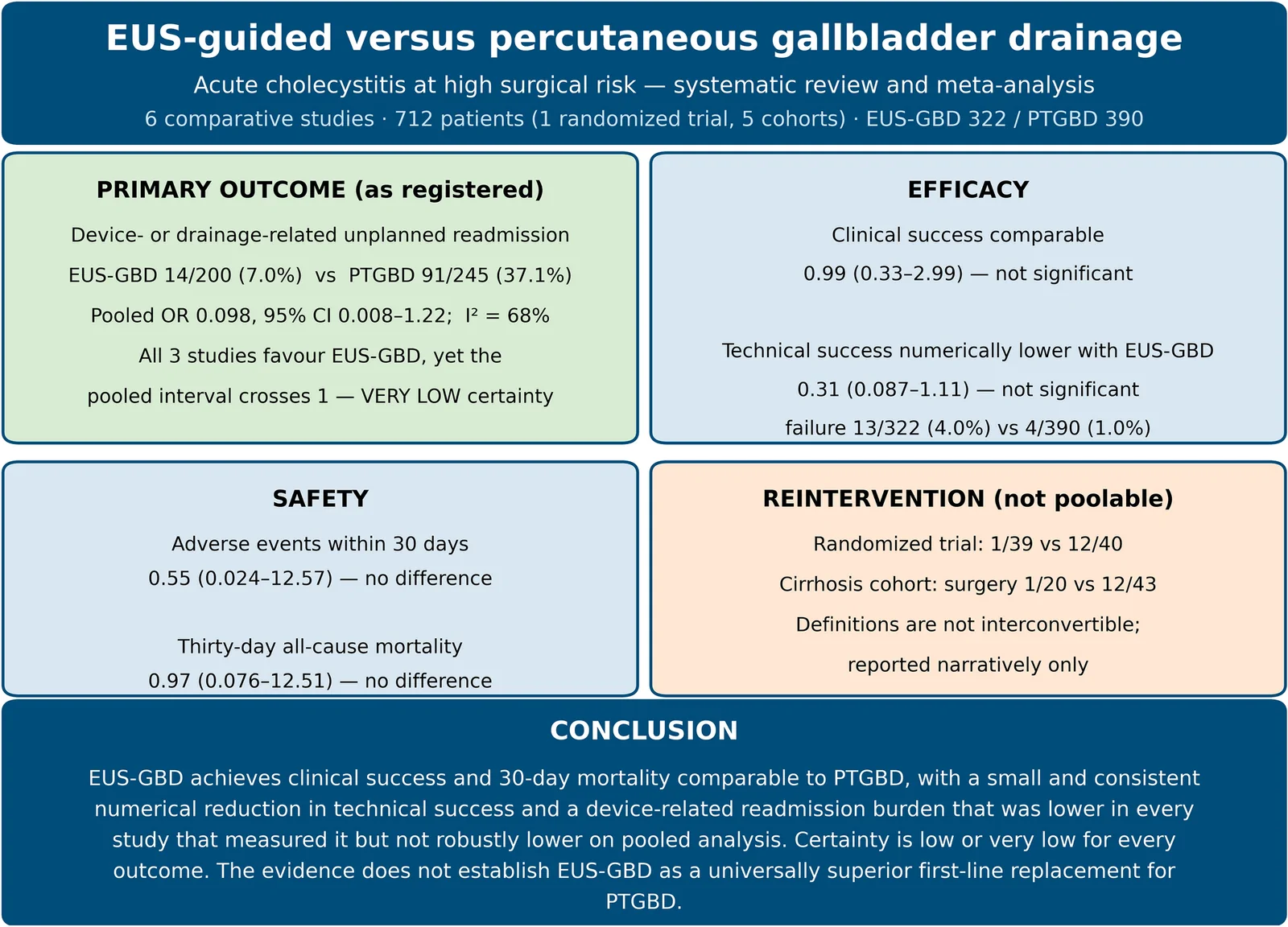

**Supplementary Information:**

The online version contains supplementary material available at https://doi.org/10.1007/s00464-026-13327-x.

Gallstone disease affects an estimated 10–20% of adults, and acute cholecystitis is among its most common complications and a leading indication for emergency abdominal surgery worldwide [1, 2]. Early laparoscopic cholecystectomy remains the standard of care; a substantial proportion of patients, however, present with advanced age, major comorbidity or physiological instability that makes early surgery prohibitively risky [1, 3, 4]. In these patients, gallbladder drainage serves as either a bridge to surgery or definitive management.

PTGBD has historically been the standard non-operative intervention in this setting. Although technically reliable and widely available, it leaves an external catheter that must be maintained, and it is associated with tube dislodgement, bile leakage, skin-site complications and recurrent device-related hospital admissions [5–9]. This burden falls disproportionately on frail and elderly patients with limited functional reserve.

EUS-GBD with LAMS creates a transmural fistula between the gallbladder and the gastric or duodenal lumen, allowing internal decompression without percutaneous access or ongoing catheter care [10–14]. Single-arm series and prospective registries report high technical success and an acceptable safety profile [15–19], and comparative data have suggested fewer device-related reinterventions with the endoscopic approach; the comparative evidence base nevertheless remains small [3, 5, 10, 11, 20].

The DRAC-1 trial—the first and, to date, only published randomized controlled trial directly comparing the two techniques—reported comparable technical success together with fewer device-related complications, unplanned admissions and reinterventions in the endoscopic arm [3]. Subsequent propensity-matched and multicentre observational studies have been directionally consistent, although heterogeneity in outcome definitions, ascertainment windows and patient selection has complicated cross-study interpretation [5, 11, 21].

Previous meta-analyses have reported comparable procedural efficacy with a possible reduction in device-related readmissions favouring EUS-GBD [6, 22–25]; a detailed methodological comparison with those syntheses is provided in the Discussion. Those analyses predate the most recent propensity-matched cohort and the first comparative study restricted to patients with cirrhosis, and an updated synthesis using small-sample-appropriate random-effects methodology is therefore warranted.

High surgical risk was defined across the included studies by one or more of the following: American Society of Anesthesiologists (ASA) physical status ≥ III, advanced age, substantial systemic comorbidity or a multidisciplinary institutional judgement that the patient was unsuitable for early cholecystectomy.

This systematic review and meta-analysis compared the effectiveness and safety of EUS-GBD using LAMS with PTGBD in surgically high-risk patients with acute cholecystitis. The primary outcome was device- or drainage-related unplanned readmission, the endpoint recorded in the public protocol registration.

## Methods

### Study design and reporting standards

This systematic review and meta-analysis was conducted and reported in accordance with the PRISMA 2020 statement [26], with the search reported according to PRISMA-S [27]. Methodological guidance was taken from the Cochrane Handbook [28].

### Protocol registration and deviations from the registered protocol

The protocol was registered on the Open Science Framework on 21 February 2026 (https://osf.io/r2ang; https://doi.org/10.17605/OSF.IO/JW9CG), before any data extraction. Because the present revision differs from the originally submitted manuscript in several respects, all deviations from the registered protocol are declared here in full.

#### Primary outcome

The registered protocol specified *unplanned or device-related readmission* as the primary outcome. The manuscript as first submitted instead reported *reintervention* as the primary outcome. During the present revision, we re-extracted every outcome from the source publications and found that reintervention cannot be pooled as a patient-level binary endpoint across these studies: Irani et al. report a count of procedures rather than of affected patients (112 procedures among 45 PTGBD patients), Pawa et al. report a median number of gallbladder interventions per patient that includes a protocol-mandated stent-removal endoscopy, and Teoh et al. (2017) and Siddiqui et al. capture reintervention only within their unplanned-admission counts [5, 10, 11, 21]. We therefore reverted to the registered endpoint. Reintervention is reported narratively. We also considered a composite of unplanned readmission *or* reintervention, but abandoned it because the patient-level union of the two components cannot be computed without double counting in any included study (Supplementary Table S5).

#### Information sources

The registered protocol specified PubMed/MEDLINE, Embase, CENTRAL and Web of Science. Embase was not accessible to the study team and was not searched; Scopus, ClinicalTrials.gov and the World Health Organization International Clinical Trials Registry Platform (WHO ICTRP) were searched in addition to the registered sources. The absence of Embase is acknowledged as a limitation.

#### Search dates

The registered protocol specified a search to March 2026. The executed search ran from inception to 31 January 2026 and was updated to 30 July 2026 for this revision; the update identified one additional eligible study [29].

#### Prediction intervals

The registered protocol proposed prediction intervals. With at most six contributing studies per outcome, the between-study variance is estimated too imprecisely for a prediction interval to be interpretable—for the primary outcome the 95% prediction interval spans more than seven orders of magnitude—and prediction intervals are therefore not reported for any outcome [30]. No inference in this manuscript rests on a prediction interval.

#### Data and code

The registered protocol stated that the dataset and code would be available on request. They are instead provided in full as downloadable supplementary files with this manuscript (Supplementary Data S1 and Supplementary Code S1) and deposited on the Open Science Framework.

### Eligibility criteria

We included randomized controlled trials and comparative observational cohort studies evaluating EUS-GBD with LAMS versus PTGBD in adults (≥ 18 years) with acute cholecystitis at high surgical risk, defined as ASA ≥ III, advanced age, substantial comorbidity burden or an institutional multidisciplinary judgement of unsuitability for early cholecystectomy. Single-arm series, non-comparative designs, case reports, narrative reviews and conference abstracts without a full-text publication were excluded. Where datasets overlapped, the most complete publication was retained. In the one three-arm study, only the EUS-GBD (transmural LAMS) and percutaneous arms were analysed; the endoscopic transpapillary arm (*n* = 124) was excluded throughout [10].

### Information sources and search strategy

PubMed/MEDLINE, Scopus, Cochrane CENTRAL and Web of Science Core Collection were searched from inception to 31 January 2026, together with ClinicalTrials.gov and the WHO ICTRP; the search was updated to 30 July 2026. Controlled vocabulary (MeSH) and free-text terms were combined across three concept blocks: acute cholecystitis; EUS-guided gallbladder drainage with LAMS; and percutaneous cholecystostomy or PTGBD. No language, publication-status or geographic restrictions were applied. Reference lists of included studies and of relevant systematic reviews were screened by hand, and forward citation tracking was performed in Web of Science and Scopus. The complete, reproducible search strings for every source, with the exact date each was run and the number of records retrieved, are given in Supplementary Appendix S1.

### Study selection and data extraction

Title/abstract screening and full-text assessment were performed independently by two investigators (R.A.G.J. and E.J.G.) using standardized forms, with disagreements resolved by consensus. Inter-rater agreement (Cohen *κ*) was 0.91 for title/abstract screening and 1.00 for full-text eligibility. Data were extracted independently by the same two investigators using a predefined form covering study design, patient demographics, procedural characteristics, verbatim outcome definitions, ascertainment windows, event counts per arm and the unit of counting (patients versus procedures), and follow-up duration. For every outcome, the denominator used was the one printed in the source article for that specific outcome, which in some studies differs from the enrolled *n*; these instances are recorded in the footnote to the table reporting the outcome concerned and, for the primary outcome, in Supplementary Table S5. The full text of one study [29] was not accessible to the study team and all of its data were taken from the indexed abstract; this is stated wherever its values appear. No imputation was performed beyond the pre-specified continuity correction for zero-event cells.

### Outcomes

The primary outcome was device- or drainage-related unplanned readmission or unplanned hospital admission, counted at the patient level, as registered. A study contributed to the primary analysis only if it reported this outcome as a count of patients with an explicit denominator. Secondary outcomes were technical success, clinical success, adverse events within 30 days and 30-day all-cause mortality. Reintervention was pre-specified as a secondary outcome but proved not to be poolable and is reported narratively. Study-level definitions, ascertainment windows, event counts, denominators and the unit of counting for the primary outcome—and the reasons for excluding each non-contributing study—are set out in full in Supplementary Table S5.

### Risk of bias assessment

The randomized trial was assessed with the Cochrane Risk of Bias 2 tool [31] and the observational studies with ROBINS-I [32]. Judgements were categorized as low, moderate, serious or critical, made independently by two investigators (R.A.G.J. and E.J.G.) using structured domain-level forms, with disagreements resolved by discussion until consensus.

### Statistical analysis

Meta-analyses were performed in R version 4.3.1 with the *meta* and *metafor* packages [33]; the complete analysis script is provided as Supplementary Code S1. Random-effects models with restricted maximum likelihood (REML) estimation of the between-study variance were used for all pooled analyses, with confidence intervals computed using the Hartung–Knapp–Sidik–Jonkman adjustment [34–36]. Because the Hartung–Knapp interval can be anticonservative when the estimated between-study variance approaches zero, the ad hoc modification of Röver, Knapp and Friede was applied throughout in its variance form: the Hartung–Knapp standard error was truncated at the random-effects (Wald) standard error, while the *t* distribution with *k*−1 degrees of freedom was retained [37]. The truncation was active for four of the six analyses—technical success, clinical success, 30-day adverse events and the post hoc device-burden sensitivity analysis—and materially changed only technical success, for which the unmodified Hartung–Knapp interval would have been 0.31 (95% CI 0.21–0.45). For the other three the change was negligible. Every interval reported in this manuscript is the modified one, because an interval narrower than the corresponding Wald interval is not defensible with six or fewer studies.

Odds ratios with 95% confidence intervals were calculated for dichotomous outcomes. For harm outcomes an OR below 1.0 favours EUS-GBD; for success outcomes an OR above 1.0 favours EUS-GBD, and the direction is stated in every table and figure. A Haldane–Anscombe continuity correction of 0.5 was applied only to studies containing a zero cell; studies with zero events in both arms were excluded from that endpoint. Because four of six studies contributed a zero cell to the technical-success analysis, that outcome was additionally re-estimated with the Mantel–Haenszel and Peto methods, which require no continuity correction.

Heterogeneity was quantified with the Cochran *Q* test, with *τ*^2^, and with the Higgins–Thompson *I*^2^ computed from *Q*, using thresholds of 25, 50 and 75% for low, moderate and high heterogeneity. Robustness was assessed by leave-one-out analysis and by re-estimation with REML plus a Wald interval, DerSimonian–Laird, Mantel–Haenszel and Peto estimators. Influence was examined with Baujat plots and Cook’s distances. Formal publication-bias testing was not performed because no outcome reached the threshold of ten studies; funnel plots are shown for visual inspection only. A two-sided *P* value below 0.05 was considered statistically significant. Odds ratios and confidence limits are reported to three decimal places below 0.10 and to two decimal places at or above 0.10, and this convention is applied identically in the text, tables and figures.

Two analyses were performed post hoc and are labelled as such wherever they appear: a sensitivity analysis adding the cirrhosis cohort’s broader long-term device-burden composite to the primary analysis (Supplementary Figure S1), and an exploratory meta-regression on coaxial double-pigtail stent use (Supplementary Table S4). Neither was pre-specified and neither informs any conclusion.

### Certainty of evidence

Certainty was assessed with GRADE across risk of bias, inconsistency, indirectness, imprecision and publication bias [38, 39], and rated as high, moderate, low or very low. Indirectness was rated not serious for all outcomes, as the populations, interventions and endpoints directly addressed the review question. A single imprecision criterion was applied identically to every pooled outcome: whether the confidence interval admits both appreciable benefit and appreciable harm. Every pooled interval does, so imprecision was rated serious throughout. Inconsistency and imprecision were rated separately because they reflect different problems—the dispersion of the study estimates and the paucity of studies—even though both follow from the small evidence base. The summary of findings is presented as Table 4.

### Reproducibility and data availability

The extracted dataset (Supplementary Data S1), the complete analysis script (Supplementary Code S1) and all supplementary materials accompany this manuscript as downloadable files and are archived on the Open Science Framework (https://osf.io/r2ang; https://doi.org/10.17605/OSF.IO/JW9CG). Every event count in the dataset carries a source field naming the table or page of the original publication from which it was taken, or, for the one study whose full text was not accessible, the indexed abstract.

## Results

### Study selection

**Fig. 1:**
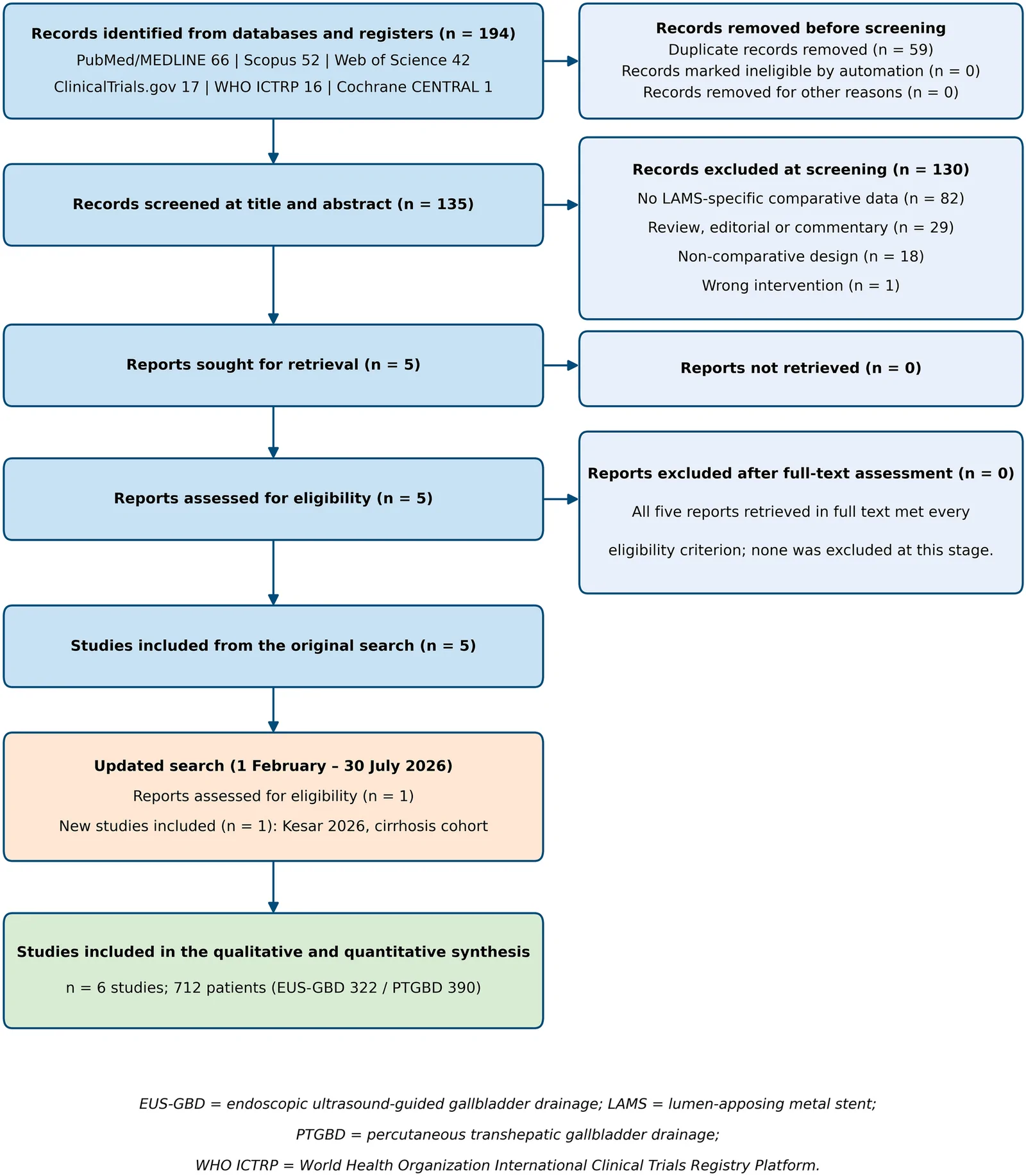
PRISMA 2020 flow diagram. Records excluded at title/abstract screening and reports excluded after full-text assessment are reported separately, with reasons. EUS-GBD, endoscopic ultrasound-guided gallbladder drainage; LAMS, lumen-apposing metal stent; PTGBD, percutaneous transhepatic gallbladder drainage; WHO ICTRP, World Health Organization International Clinical Trials Registry Platform

The PRISMA 2020 flow diagram is presented as Fig. 1. The original search identified 194 records (PubMed/MEDLINE 66, Scopus 52, Web of Science 42, ClinicalTrials.gov 17, WHO ICTRP 16, Cochrane CENTRAL 1). After 59 duplicates were removed, 135 records were screened at title and abstract and 130 were excluded, the commonest reasons being absence of LAMS-specific comparative data (82) and review or editorial article type (29). Five reports were sought for retrieval, all five were retrieved and assessed in full text, and none was excluded at that stage. The updated search identified one further eligible comparative study [29]. Six studies comprising 712 patients (EUS-GBD 322; PTGBD 390) were included in the qualitative and quantitative synthesis.

### Study characteristics and risk of bias

**Fig. 2:**
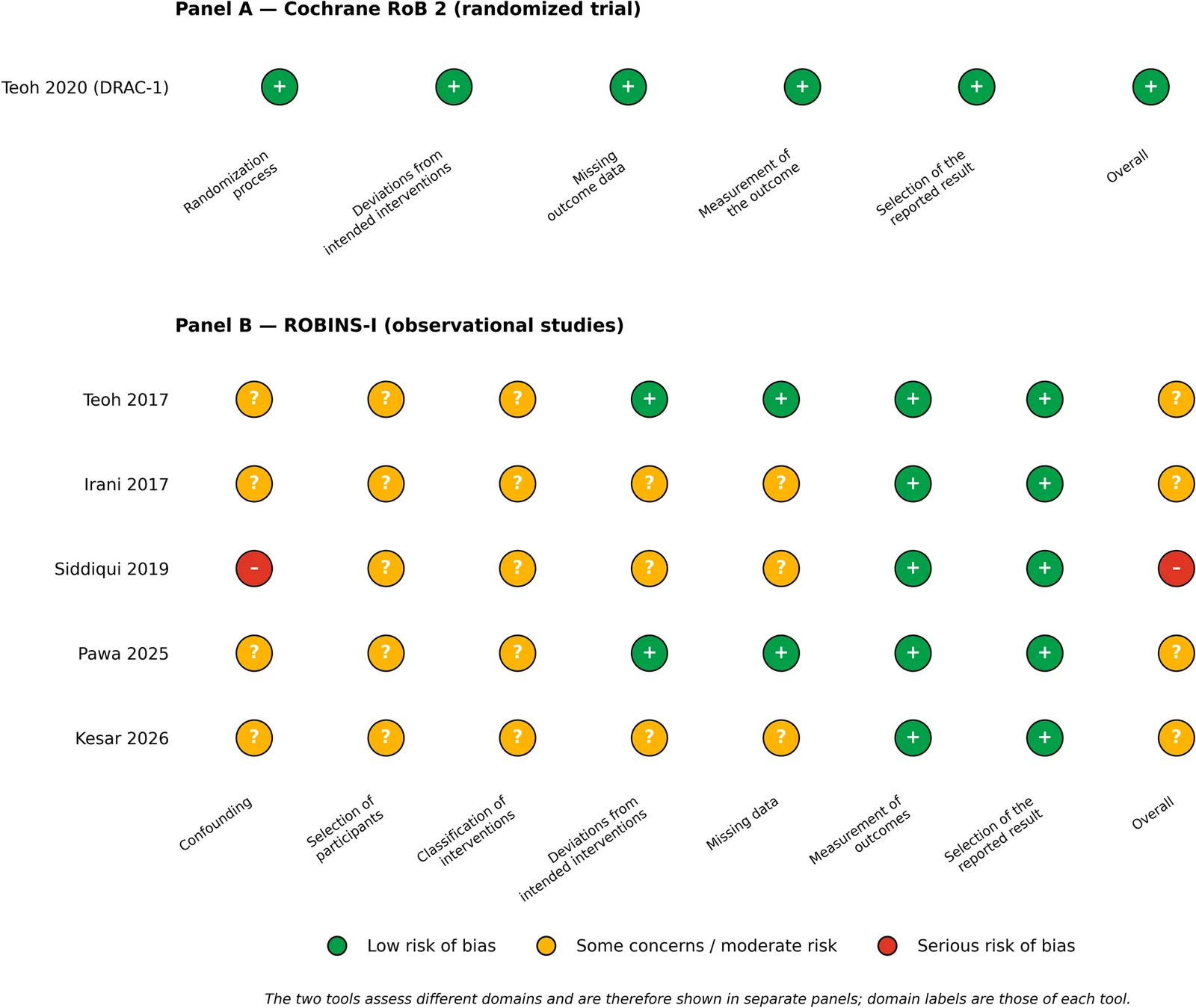
Risk of bias. Panel **A**, Cochrane RoB 2 applied to the randomized trial [3]: low risk in every domain and low risk overall. Panel **B**, ROBINS-I applied to the five observational studies: four at overall moderate risk, and the three-arm non-randomized study at serious risk for confounding and therefore serious overall [10]. The two tools assess different domains and are shown in separate panels. Green (+), low risk; amber (?), moderate risk or some concerns; red (–), serious risk

Characteristics of the included studies are given in Table 1. One study was a randomized controlled trial [3] and five were comparative observational studies: a propensity score-matched cohort [11], a matched cohort [21], two multicentre retrospective analyses [5, 10] and a multicentre retrospective cohort restricted to patients with cirrhosis [29]. Follow-up ranged from a median of 3–4 months [10] to a mean exceeding 800 days in the PTGBD arm of one matched cohort [21]. The randomized trial was judged at low risk of bias by RoB 2. Four observational studies were rated at overall moderate risk by ROBINS-I; the three-arm non-randomized study was rated serious for confounding and therefore serious overall, because ROBINS-I aggregates to the most severe domain judgement [10] (Fig. 2).

**Table 1:** Characteristics of the six included comparative studies (*n* = 712; EUS-GBD 322/PTGBD 390)

Study	Year	Design	EUS-GBD/PTGBD (*n*)	Population and high-risk criterion	Calculous/malignant	Follow-up	Risk of bias
Teoh (DRAC-1)	2020	Randomized controlled trial, multicentre	39/40	ASA ≥ III, very high surgical risk	100% (calculous only by design)/0%	All patients followed to 1 year or death; 0 lost to follow-up	Low (RoB 2)
Teoh	2017	Matched cohort, 2 centres	59/59	Tokyo 2013, ASA ≥ III, unfit for surgery	100% (calculous only by design)/NR	Mean 451 (SD 343) vs. 834 (SD 417) days; *P* < .001	Moderate (ROBINS-I)
Irani	2017	Multicentre retrospective cohort, matched	45/45	ASA IV–V predominant; widespread malignancy	60.0% vs. 75.6%/35.6% vs. 20.0%	Median 215 (range 1–621) vs. 265 (range 1–1638) days	Moderate (ROBINS-I)
Siddiqui	2019	Multicentre retrospective, three-arm	102/146	ASA IV–V; not fit for cholecystectomy	73.7% vs. 68.0%/18.4% vs. 15.0%	Median 4 (IQR 3–9) vs. 3 (IQR 3–4) months; minimum 3 months	Serious (ROBINS-I; confounding)
Pawa	2025	Retrospective propensity score-matched (1:1)	57/57	ASA, ECOG and Charlson comorbidity index	70% in both arms/30% vs. 16%	Median 488 vs. 692 days; *P* = .11	Moderate (ROBINS-I)
Kesar	2026	Multicentre retrospective cohort (cirrhosis)	20/43	Cirrhosis, unfit for surgery	63%/NR (37% acalculous)	Mean 19.9 weeks (range 1–52), cohort overall	Moderate (ROBINS-I)

Values are given as EUS-GBD versus PTGBD wherever the source article reports them by arm, and for the cohort as a whole otherwise

Kesar 2026 was identified by the search update; the full text was not accessible and all of its data come from the indexed abstract (PMID 42298032), which reports follow-up and aetiology for the cohort as a whole rather than by arm. In the three-arm study, only the EUS-GBD (transmural LAMS) and percutaneous arms were analysed; the endoscopic transpapillary arm (*n* = 124) was excluded throughout

*ASA* American Society of Anesthesiologists physical status, *ECOG* Eastern Cooperative Oncology Group, *EUS-GBD* endoscopic ultrasound-guided gallbladder drainage, *IQR* interquartile range, *NR* not reported, *PTGBD* percutaneous transhepatic gallbladder drainage, *SD* standard deviation

### Baseline characteristics

Baseline demographic and clinical characteristics are presented in Supplementary Table S1, arm by arm wherever the source article reports them that way. Mean age across arms ranged from 60.4 years in the percutaneous arm of the three-arm study [10] to 82.7 years in the endoscopic arm of one matched cohort [21]; that three-arm study was the only one with a significant between-arm age difference (72.4 versus 60.4 years, *P* < 0.001). The proportion of male patients ranged from 50.0 to 64.4%. Calculous disease accounted for 60.0 to 100% of cases and was an inclusion criterion in both Teoh cohorts [3, 21]; the cirrhosis cohort had the highest proportion of acalculous disease (37%) [29]. Underlying malignancy, where reported, ranged from 15.0 to 35.6%; it was 0% by design in the randomized trial and was not reported in two studies. Severity grading was not uniformly reported and the categories used are not interchangeable: two studies report Tokyo grade II–III (100, and 86.7% versus 82.2%), while one reports grade I–II (91% versus 88%), so no single range can be quoted (Supplementary Table S2) [2, 40].

### Ascertainment of the primary outcome

Only three of the six studies reported device- or drainage-related unplanned readmission as a count of patients with an explicit denominator [3, 10, 21]. In each, the printed percentages are computed on patient denominators, confirming that the unit of counting is patients rather than admission episodes; two of the three state explicitly that some patients had more than one admission, which the tabulated counts do not double-count [10, 21]. The remaining three studies could not contribute: one reports reinterventions only as a count of procedures and gives no EUS-GBD readmission figure [5], one does not use the concept of readmission anywhere in the article [11], and the cirrhosis cohort reports a long-term adverse-event composite rather than readmission [29]. Because the primary endpoint has a single component, no patient can be counted twice within it. Verbatim definitions, ascertainment windows, event counts, denominators, the unit of counting and the reason for each exclusion are tabulated study by study in Supplementary Table S5.

### Primary outcome: device- or drainage-related unplanned readmission

**Fig. 3:**
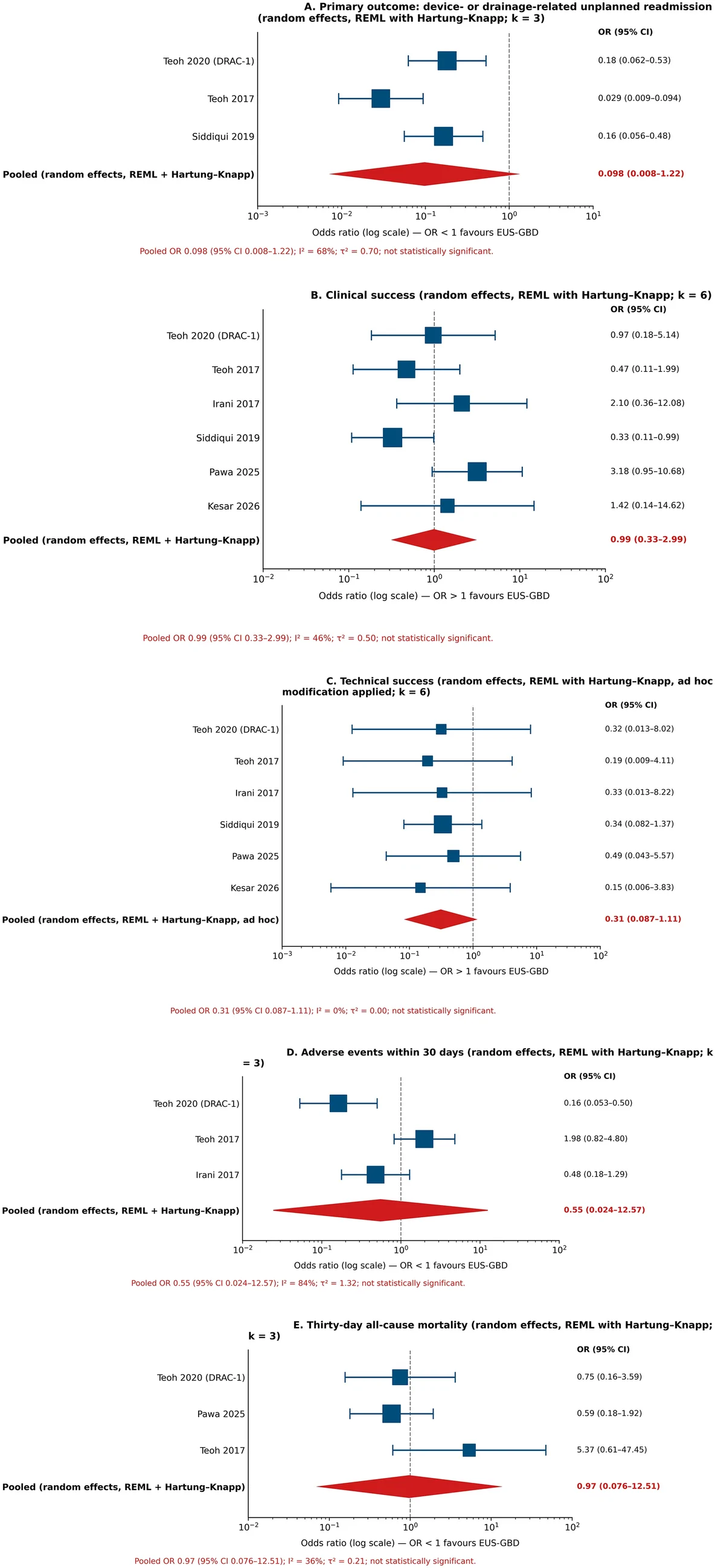
**A** Primary outcome: device- or drainage-related unplanned readmission (random-effects REML with the Hartung–Knapp adjustment; *k* = 3). An odds ratio below 1 favours EUS-GBD. Pooled OR 0.098, 95% CI 0.008–1.22; *I*^2^ = 68%. Each contributing interval excludes unity; the pooled interval does not. **B** Clinical success (random-effects REML with the Hartung–Knapp adjustment; *k* = 6). An odds ratio above 1 favours EUS-GBD. Pooled OR 0.99, 95% CI 0.33–2.99; *I*^2^ = 46%; not statistically significant. **C** Technical success (random-effects REML with the Hartung–Knapp adjustment and the variance form of its ad hoc modification; *k* = 6). An odds ratio above 1 favours EUS-GBD. Pooled OR 0.31, 95% CI 0.087–1.11; *I*^2^ = 0%; the interval includes unity. Because the estimated between-study variance was zero, the Hartung–Knapp standard error was truncated at the Wald standard error; the unmodified interval would have been 0.31 (0.21–0.45). **D** Adverse events within 30 days (random-effects REML with the Hartung–Knapp adjustment; *k* = 3). An odds ratio below 1 favours EUS-GBD. Pooled OR 0.55, 95% CI 0.024–12.57; *I*^2^ = 84%; not statistically significant. **E** Thirty-day all-cause mortality (random-effects REML with the Hartung–Knapp adjustment; *k* = 3). An odds ratio below 1 favours EUS-GBD. Pooled OR 0.97, 95% CI 0.076–12.51; *I*^2^ = 36%; not statistically significant

Device- or drainage-related unplanned readmission occurred in 14/200 (7.0%) of EUS-GBD patients and 91/245 (37.1%) of PTGBD patients across the three contributing studies (*n* = 445). Each study favoured EUS-GBD and each study-level confidence interval excluded unity: 6/39 versus 20/40 (OR 0.18, 95% CI 0.062–0.53) in the randomized trial [3]; 4/59 versus 42/59 (OR 0.029, 95% CI 0.009–0.094) [21]; and 4/102 versus 29/146 (OR 0.16, 95% CI 0.056–0.48) [10]. The pooled estimate was OR 0.098, 95% CI 0.008–1.22; *P* = 0.058; *I*^2^ = 68%; *τ*^2^ = 0.70; *Q* = 6.28 (df = 2, *P* = 0.043) (Fig. 3A). The Hartung–Knapp interval therefore includes unity even though every contributing interval excludes it—a consequence of applying a deliberately conservative small-sample adjustment to only three studies whose effect magnitudes differ roughly six-fold.

This divergence between the individual studies and the pooled interval is the central quantitative finding of the review and is the reason the primary result is not interpreted as demonstrating superiority. With alternative estimators that do not apply the small-sample adjustment the interval excludes unity—REML with a Wald interval 0.098 (0.031–0.31), DerSimonian–Laird 0.098 (0.032–0.30), Mantel–Haenszel 0.10 (0.054–0.19) and Peto 0.15 (0.093–0.23)—but these are reported only to show that the point estimate is stable, not to supersede the pre-specified analysis.

### Sensitivity and robustness analyses for the primary outcome

Leave-one-out results are given in Supplementary Table S4. Omission of the randomized study leaves only two, and a Hartung–Knapp interval with one degree of freedom is not interpretable, so no leave-one-out fit yields a usable interval. The point estimates remain in the same direction throughout (0.071, 0.17 and 0.074), and heterogeneity disappears when the matched cohort with the most extreme estimate is omitted (*I*^2^ = 0%), which identifies that study as the source of the dispersion. The pooled estimate is therefore not robust to the removal of any single study, and this fragility is reflected in the GRADE rating.

In a post hoc sensitivity analysis, we added the cirrhosis cohort using its broader long-term composite of biliary tract-related and catheter- or device-related adverse events (2/19 versus 18/43) [29]. This is a different construct from unplanned readmission and the analysis is exploratory, but it is directionally identical and, with four studies, reaches statistical significance (OR 0.11, 95% CI 0.025–0.46; *I*^2^ = 54%; Supplementary Figure S1).

### Technical success

Technical success was high with both techniques but consistently lower with EUS-GBD: 309/322 (96.0%) versus 386/390 (99.0%) (per-study range 94.1–97.8% for EUS-GBD and 97.9–100% for PTGBD). In absolute terms, technical failure occurred in 13/322 (4.0%) of EUS-GBD procedures versus 4/390 (1.0%) of PTGBD procedures. The pooled odds ratio for technical success was OR 0.31, 95% CI 0.087–1.11 (*P* = 0.065; *I*^2^ = 0%; Fig. 3C), an interval that includes unity. Heterogeneity was absent, so the estimated between-study variance was zero and the ad hoc truncation was active here; the unmodified Hartung–Knapp interval would have been 0.31 (0.21–0.45) and is not reported, being narrower than the corresponding Wald interval. Four of six studies contributed a zero cell, so the analysis was repeated with the two estimators that require no continuity correction, and both excluded unity (Mantel–Haenszel 0.23 (0.074–0.72); Peto 0.26 (0.098–0.68)). The direction is thus consistent in all six studies and across estimators, but the pre-specified small-sample-adjusted interval does not establish a difference. Where a reason was reported, the commonest cause of EUS-GBD failure was the absence of an identifiable enteral puncture site, accounting for 8 of the 13 failures and for all six in the largest cohort [3, 10, 21]; the other four were device-related, namely stent misdeployment or migration [5, 11, 21], and the reason for the single remaining failure was not reported [29]. Three of the four PTGBD failures were attributed to a small, stone-filled gallbladder lumen that would not accommodate the catheter [10].

### Clinical success

Clinical success was comparable between techniques: 295/321 (91.9%) versus 360/389 (92.5%) (OR 0.99, 95% CI 0.33–2.99; *P* = 0.985; *I*^2^ = 46%; Fig. 3B). Moderate heterogeneity is consistent with the substantial variation in how clinical success was defined and when it was assessed, which ranged from three days to five days, with one study using a 96-h window that also carried a recurrence clause with no time limit and one study stating no window at all; the study-level definitions are set out in Table 2.

**Table 2:** Study-level operational definitions of clinical success

Study	Definition as reported	Ascertainment window	EUS-GBD	PTGBD
Teoh 2020 (DRAC-1)	Improvement in clinical symptoms and laboratory test	Not stated (protocol checked tube/stent patency at 96 h)	36/39 (92.3%)	37/40 (92.5%)
Teoh 2017	Improvement in clinical symptoms and decreasing white cell count	Within 5 days	53/59 (89.8%)	56/59 (94.9%)
Irani 2017	Resolution of symptoms and of laboratory and/or radiological abnormalities	Within 3 days	43/45 (95.6%)	41/45 (91.1%)
Siddiqui 2019	Afebrile, resolution of leukocytosis and of abdominal pain, and able to tolerate oral intake	Within 5 days (a conflicting 72-h definition also appears in the same Methods section)	92/102 (90.2%)	141/146 (96.6%)
Pawa 2025	Resolution of abdominal pain and fever with normalization of the white cell count, without recurrence of cholecystitis	96 h for the symptom and laboratory components; the recurrence clause is not time-bounded	52/56 (92.9%)	45/56 (80.4%)
Kesar 2026	Not defined in the available record; the abstract states only that clinical success was reported on an intention-to-treat and a per-protocol basis	Not stated in the available record	19/20 (95.0%)	40/43 (93.0%)

Definitions and ascertainment windows differ materially across studies; this variation is the most likely source of the moderate heterogeneity observed for this outcome (*I*^2^ = 46%)

Pawa 2025 prints a denominator of 56 rather than 57 in both arms for this outcome; the printed denominator has been used, as it reproduces the *P* value reported in that article

### Adverse events within 30 days

Three studies reported adverse events using a 30-day window and graded them with the Clavien–Dindo classification or the American Society for Gastrointestinal Endoscopy lexicon [3, 5, 21, 41]. Pooled, they did not differ: 30/143 (21.0%) versus 43/144 (29.9%) (OR 0.55, 95% CI 0.024–12.57; *P* = 0.502; *I*^2^ = 84%; Fig. 3D). Heterogeneity was the highest of any pooled outcome, and the study estimates fell on opposite sides of unity by a wide margin: the randomized trial strongly favoured EUS-GBD (OR 0.16, 95% CI 0.053–0.50), whereas the matched cohort favoured PTGBD (OR 1.98, 95% CI 0.82–4.80); the latter recorded three intraprocedural events, all within the institution’s first 20 EUS-GBD procedures [21]. Influence diagnostics identified no single dominant study (Supplementary Figures S3 and S5), supporting genuine between-study variability rather than a single outlier. Three studies could not contribute because their adverse-event windows differ from 30 days: a 7-day procedural window [10], an undifferentiated window with no stated horizon [11], and procedure-related events with no stated window [29]. One caveat applies to the analysis as performed. Although Irani et al. define their adverse-event outcome as events occurring within 30 days, at least four of the eight EUS-GBD events and three of the fourteen PTGBD events reported in that article are dated beyond 30 days and the remainder are undated, so a strictly 30-day count cannot be reconstructed from the publication [5]. The study is retained because 30 days is the window its authors specify, and this is a further reason not to over-interpret this outcome (Table 3).

**Table 3:** Pooled outcomes

Outcome	*k*	EUS-GBD (events/total)	PTGBD (events/total)	OR (95% CI)	*P*	*I*^2^ (%)	Direction favouring EUS-GBD	Result
Device- or drainage-related unplanned readmission (PRIMARY)	3	14/200 (7.0%)	91/245 (37.1%)	0.098 (0.008–1.22)	.058	68	OR < 1	Not significant
Technical success	6	309/322 (96.0%)	386/390 (99.0%)	0.31 (0.087–1.11)	.065	0	OR > 1	Not significant
Clinical success	6	295/321 (91.9%)	360/389 (92.5%)	0.99 (0.33–2.99)	.985	46	OR > 1	Not significant
Adverse events within 30 days	3	30/143 (21.0%)	43/144 (29.9%)	0.55 (0.024–12.57)	.502	84	OR < 1	Not significant
Thirty-day all-cause mortality	3	13/154 (8.4%)	13/155 (8.4%)	0.97 (0.076–12.51)	.968	36	OR < 1	Not significant
Post hoc sensitivity: broader long-term device-burden composite, adding the cirrhosis cohort	4	16/219 (7.3%)	109/288 (37.8%)	0.11 (0.025–0.46)	.016	54	OR < 1	Significant (post hoc, exploratory)
Reintervention after 30 days—randomized trial only, narrative	1	1/39 (2.6%)	12/40 (30.0%)	0.06 (0.008–0.50)	.001	–	OR < 1	Favours EUS-GBD (single trial; not pooled)

Random-effects restricted maximum likelihood models with the Hartung–Knapp–Sidik–Jonkman adjustment

The direction column states explicitly which side of unity favours EUS-GBD, because this differs between harm outcomes and success outcomes

Primary outcome: every contributing study favoured EUS-GBD and every study-level interval excluded unity, but the pooled Hartung–Knapp interval does not; the estimate is not robust to leave-one-out omission (Supplementary Table S4)

Technical success: the estimated between-study variance was zero, so the variance form of the ad hoc modification was active and the Hartung–Knapp standard error was truncated at the Wald standard error; the unmodified Hartung–Knapp interval would have been 0.31 (0.21–0.45) and is not reported because it is narrower than the Wald interval. Four of six studies contributed a zero cell, so the analysis was repeated with the two estimators requiring no continuity correction, both of which exclude unity: Mantel–Haenszel 0.23 (0.074–0.72), Peto 0.26 (0.098–0.68). The direction is consistent in all six studies, but the pre-specified interval does not establish a difference

Adverse events within 30 days: Irani et al. define this outcome as events occurring within 30 days, but at least four of their eight EUS-GBD events and three of their fourteen PTGBD events are dated beyond 30 days and the remainder are undated (Supplementary Table S4)

Post hoc sensitivity row: the EUS-GBD denominator of 219 is 200 + 19. The cirrhosis cohort contributes 19 of its 20 EUS-GBD patients to that outcome because its long-term adverse-event percentages are computed on the 19 patients in whom the procedure was technically successful (10.5% = 2/19); the denominator itself is not printed in the available record and was derived from that percentage

Reintervention was not pooled: the constructs are not interconvertible across studies (see Results, narrative synthesis, and Supplementary Table S5)

Odds ratios and confidence limits are given to three decimal places below 0.10 and to two decimal places at or above 0.10, identically in the text, tables and figures

*CI* confidence interval, *k* number of contributing studies, *OR* odds ratio

### Thirty-day all-cause mortality

Thirty-day all-cause mortality did not differ significantly in the three studies that reported it [3, 11, 21]: 13/154 (8.4%) versus 13/155 (8.4%) (OR 0.97, 95% CI 0.076–12.51; *P* = 0.968; *I*^2^ = 36%; Fig. 3E). The observed proportions were the same in the two arms, but the interval accommodates a large difference in either direction, so this is an absence of evidence rather than evidence of equivalence. The three remaining studies did not report 30-day all-cause mortality: two report only procedure-related deaths or deaths without a time horizon [5, 10] and one does not report it [29].

### Reintervention (narrative synthesis)

Reintervention could not be pooled because the studies do not use a common unit or a common construct. In the randomized trial, reintervention after 30 days occurred in 1/39 (2.6%) vs. 12/40 (30.0%); OR 0.06, 95% CI 0.008–0.50 [3]. In the cirrhosis cohort, subsequent surgery was required in 1/20 (5%) vs. 12/43 (28%); *P* = 0.03, and 7 of the 12 PTGBD operations were urgent procedures driven by drainage failure [29]. One cohort reported 11 versus 112 reintervention *procedures* (0.2 ± 0.4 versus 2.5 ± 2.8 per patient) without stating how many patients were affected [5]; one reported a median of 2 versus 3 total gallbladder interventions per patient, a count that includes the protocol-mandated LAMS-removal endoscopy in the EUS-GBD arm [11]; and two capture reintervention only inside their unplanned-admission counts [10, 21]. Every study that reports the outcome in any form favours EUS-GBD, but the constructs are not interconvertible and no pooled estimate is defensible.

### Certainty of evidence

GRADE assessments are presented in Table 4. No outcome was rated above low certainty. The primary outcome and 30-day adverse events were rated very low, each downgraded for risk of bias, for inconsistency and for imprecision. Technical success, clinical success, 30-day mortality and the patient-level reintervention estimate from the single randomized trial were rated low. A single imprecision criterion—whether the interval admits both appreciable benefit and appreciable harm—was applied identically to every pooled outcome.

**Table 4:** GRADE summary of findings

Outcome	*k*	*n*	Effect (OR, 95% CI)	Risk of bias	Inconsistency	Indirectness	Imprecision	Certainty
Device- or drainage-related unplanned readmission (primary)	3	445	0.098 (0.008–1.22)	Serious	Serious (*I*^2^ = 68%; roughly six-fold spread of effect magnitudes)	Not serious	Serious (interval admits both appreciable benefit and appreciable harm; only three studies contribute)	⊕◯◯◯ Very low
Technical success	6	712	0.31 (0.087–1.11)	Serious	Not serious (*I*^2^ = 0%)	Not serious	Serious (17 events in total; interval includes unity; optimal information size not met)	⊕⊕◯◯ Low
Clinical success	6	710	0.99 (0.33–2.99)	Serious	Not serious (*I*^2^ = 46%)	Not serious	Serious (interval includes both appreciable benefit and appreciable harm)	⊕⊕◯◯ Low
Adverse events within 30 days	3	287	0.55 (0.024–12.57)	Serious	Serious (*I*^2^ = 84%; direction of effect reverses between studies)	Not serious	Serious (interval spans more than two orders of magnitude)	⊕◯◯◯ Very low
Thirty-day all-cause mortality	3	309	0.97 (0.076–12.51)	Serious	Not serious (*I*^2^ = 36%)	Not serious	Serious (26 events in total; interval admits both appreciable benefit and appreciable harm)	⊕⊕◯◯ Low
Reintervention after 30 days (randomized trial only)	1	79	0.06 (0.008–0.50)	Not serious (low risk of bias)	Not applicable (single study)	Not serious	Very serious (single trial, 13 events; optimal information size not met)	⊕⊕◯◯ Low

a. All bodies of evidence start at high certainty because a randomized trial contributes to each pooled outcome, and are downgraded from there. Risk of bias was rated serious for every pooled outcome because five of six studies are observational and one of those is at overall serious risk of bias under ROBINS-I, driven by confounding

b. No outcome was rated above low certainty, and no outcome was upgraded

c. A single imprecision criterion was applied to every pooled outcome: whether the confidence interval admits both appreciable benefit and appreciable harm. Every pooled interval does, so imprecision is rated serious throughout. For the primary outcome the width is driven mainly by the *t* quantile with two degrees of freedom (4.30 rather than 1.96) that follows from having only three contributing studies; the optimal information size for a binary outcome is not met at 105 events. Inconsistency and imprecision are rated separately here because they reflect distinct problems—dispersion of the study estimates, and the paucity of studies—although both stem from the small evidence base

d. Publication bias could not be assessed for any outcome because none reached ten studies; it was rated undetected rather than not serious, and no outcome was downgraded on this domain

*CI* confidence interval, *k* number of contributing studies, *n* number of patients contributing to that outcome, *OR* odds ratio

## Discussion

In surgically high-risk patients with acute cholecystitis, this synthesis of six comparative studies and 712 patients found that EUS-GBD with LAMS achieves clinical success and 30-day mortality that do not differ significantly from PTGBD, with a small and consistent numerical reduction in technical success, and with a device-related readmission burden that was lower in every study that measured it. Neither the primary estimate nor the technical-success estimate excluded unity under the pre-specified small-sample-adjusted model, and certainty is low or very low for every outcome.

The most informative feature of the primary analysis is the gap between the study-level and the pooled inference. In all three contributing studies, the odds ratio favoured EUS-GBD and the study-level confidence interval excluded unity, with point estimates of 0.18, 0.029 and 0.16; yet with only three studies and a six-fold spread of magnitudes, the Hartung–Knapp adjustment yields an interval of 0.008–1.22. This is the intended behaviour of that adjustment, which protects against the well-documented anticonservatism of conventional random-effects intervals when the number of studies is small [34–36]. It should be read as a statement about how little the current literature constrains the size of the effect, not as evidence that no effect exists. The direction is consistent and mechanistically plausible: EUS-GBD leaves no external catheter to dislodge, block, leak or become infected, and catheter-related problems account for the great majority of unplanned admissions in the percutaneous arms [7–9].

Our estimates are directionally concordant with earlier syntheses of this comparison [6, 22–25] and with a recent network meta-analysis [42], but our intervals are wider. That is a consequence of the estimator rather than of the data: conventional DerSimonian–Laird intervals are known to be too narrow when few studies are pooled [36], and our own DerSimonian–Laird re-estimation of the primary outcome (0.098 (0.032–0.30)) reproduces the narrower intervals reported previously. We regard the wider intervals as a more faithful representation of a sparse evidence base rather than as a contradiction of earlier work.

Technical success deserves comment because it is usually presented as equivalent between the two techniques. Technical failure was about four times more common with EUS-GBD (13/322 (4.0%)) than with PTGBD (4/390 (1.0%)); the direction was the same in all six studies and there was no heterogeneity at all (*I*^2^ = 0%). We are nevertheless careful not to overstate this. Under the pre-specified small-sample-adjusted model the interval includes unity (OR 0.31, 95% CI 0.087–1.11), and only the estimators that require no continuity correction exclude it (0.23 (0.074–0.72) and 0.26 (0.098–0.68)); four of six studies contributed a zero cell, so the pooled odds ratio depends heavily on the continuity correction. The defensible statement is the absolute one: roughly one EUS-GBD procedure in twenty-five fails technically, against roughly one PTGBD procedure in a hundred. That difference is small but clinically interpretable, because 8 of the 13 EUS-GBD failures were attributable to the absence of an identifiable enteral puncture site, an anatomical constraint that can be anticipated on pre-procedural imaging and that PTGBD does not share; the other four were stent misdeployment or migration, a failure mode that would be expected to fall with operator experience. Percutaneous drainage therefore retains a defined role, and pre-procedural cross-sectional imaging to establish whether a transenteric window exists is a reasonable prerequisite for offering EUS-GBD.

Adverse events within 30 days did not differ, and this was the only outcome in which the direction of effect reversed between studies (*I*^2^ = 84%). The reversal is attributable to one matched cohort in which all three intraprocedural events occurred within the institution’s first twenty EUS-GBD procedures [21]. This is a learning-curve signal rather than a property of the technique, and it is consistent with registry data showing that technical and safety outcomes improve with operator experience [18]. It also means that the pooled adverse-event estimate should not be extrapolated to centres at either extreme of procedural volume.

Clinical heterogeneity across the included studies was substantial and warrants caution on six specific axes. Calculous disease ranged from 60 to 100%, with the cirrhosis cohort containing 37% acalculous disease [29]. Underlying malignancy ranged from 15.0 to 35.6% where reported, and was 0% by design in the randomized trial [3]. Follow-up ranged from a median of three months to a mean beyond 800 days, and in one matched cohort the percutaneous arm was followed almost twice as long as the endoscopic arm, which the original authors identified as a source of lead-time bias inflating the percutaneous event rate [21]. LAMS platforms and the use of electrocautery-enhanced delivery differed. Coaxial double-pigtail stenting was systematic in one study, selective in another and unstated elsewhere [5, 11]. Finally, the timing and feasibility of subsequent cholecystectomy differed; interval cholecystectomy after EUS-GBD appears feasible with surgical outcomes comparable to those after PTGBD [43, 44].

Ascertainment windows are the single most important source of non-comparability in this literature and the principal obstacle to pooling. Adverse events were captured over 7 days in one study, 30 days in three and an unbounded interval in another; readmissions were captured over 12 months in one study and over the whole of a variable follow-up in others. Short windows systematically under-capture late device-related events, which are precisely the events that distinguish an internal from an external drain. Until comparative studies adopt a common, patient-level, time-anchored definition of device-related burden, meta-analysis of this endpoint will remain limited by definitional heterogeneity rather than by sample size.

Strengths of this review include duplicate independent screening and extraction with excellent agreement; re-extraction of every event count directly from the source publications, with the unit of counting and the ascertainment window recorded for each; the use of REML with the Hartung–Knapp adjustment and its ad hoc modification throughout [36]; concordance checks against four alternative estimators; formal GRADE assessment [38, 39]; and public availability of the dataset and the analysis code in two independent implementations, so that every number in this manuscript can be recomputed.

Several limitations should be weighed. Five of six studies were observational and residual confounding by indication cannot be excluded, since the choice between techniques is rarely random in practice. Only three studies could contribute to the primary outcome, and only three to each of the adverse-event and mortality analyses. Embase was not searched, which may have reduced sensitivity; this is partly mitigated by searching Scopus and Web of Science, whose coverage overlaps substantially with Embase, and by hand-searching reference lists and forward citations, but it remains a deviation from the registered protocol. Most data derive from high-volume academic centres with advanced endoscopic expertise, limiting generalizability to lower-volume settings. Publication bias could not be assessed formally. Finally, no included study reported patient-reported outcomes or cost, both of which are central to a decision between an internal and an external drain in a frail population.

For practice, these data support EUS-GBD as a reasonable internal-drainage option where advanced endoscopic expertise is available and a transenteric window exists, particularly for patients in whom prolonged external catheter dependence is undesirable. They do not support replacing PTGBD as the default. For research, the priority is an adequately powered randomized trial using a single, pre-specified, patient-level device-burden endpoint with a fixed ascertainment window, alongside patient-reported outcomes and formal cost analysis; a trial sequential analysis has already indicated that the accumulated evidence falls short of the required information size [45]. Long-term follow-up beyond one year, where the advantage of internal drainage would be expected to be largest, remains particularly sparse [46].

## Conclusion

In surgically high-risk patients with acute cholecystitis, EUS-GBD with lumen-apposing metal stents achieved clinical success and 30-day mortality comparable to PTGBD, with a small and consistent numerical reduction in technical success (failure 13/322 (4.0%) versus 4/390 (1.0%)) and with a device- or drainage-related readmission burden that was lower in every study that measured it (7.0 versus 37.1%) but not robustly lower on pooled analysis (OR 0.098, 95% CI 0.008–1.22). Certainty is low or very low for every outcome. EUS-GBD is a reasonable internal-drainage option where advanced endoscopic expertise and a suitable transenteric window are available, but the present evidence does not establish it as a universally superior or first-line replacement for PTGBD. Adequately powered randomized trials with standardized, patient-level outcome definitions, fixed ascertainment windows, extended follow-up, patient-reported outcomes and cost analyses are required.

## Supplementary Information

Below is the link to the electronic supplementary material.

Supplementary file1 (DOCX 2119 KB)

## Data Availability

The extracted dataset (Supplementary Data S1) and the complete analysis code (Supplementary Code S1) are provided as downloadable supplementary files and are archived on the Open Science Framework (https://osf.io/r2ang; https://doi.org/10.17605/OSF.IO/JW9CG).

## Declarations

### Protocol registration

Open Science Framework https://osf.io/r2ang (https://doi.org/10.17605/OSF.IO/JW9CG), registered 21 February 2026.
