# Supplementary material for: Endoscopic ultrasound-guided versus percutaneous transhepatic gallbladder drainage in surgically high-risk patients with acute cholecystitis: a systematic review and meta-analysis
Source: Surg Endosc. 2026 Sep 22;40(10):8367–82. doi: 10.1007/s00464-026-13327-x (PMC13633151; doi:10.1007/s00464-026-13327-x)
Supplement: Supplementary file 1 [file 464_2026_13327_MOESM1_ESM.docx]

**Supplementary Material**

*Endoscopic Ultrasound-Guided Versus Percutaneous Transhepatic Gallbladder Drainage in Surgically High-Risk Patients with Acute Cholecystitis: A Systematic Review and Meta-Analysis*

Manuscript SEND-D-26-00521, Surgical Endoscopys

## Contents

• Supplementary Table S0. Completed PRISMA 2020 checklist

• Supplementary Appendix S1. Full search strategy

• Supplementary Table S1. Baseline demographic and clinical characteristics

• Supplementary Table S2. Pre-procedural severity and laboratory profile

• Supplementary Table S3. EUS-GBD technical protocol and device configuration

• Supplementary Table S4. Sensitivity, robustness and post hoc exploratory analyses

• Supplementary Table S5. Study-level ascertainment of the primary outcome

• Supplementary Figures S1–S7

• Supplementary Data S1 (separate file: extracted dataset, .xlsx and .csv)

• Supplementary Code S1 (separate files: R analysis script and Python pipeline)

# Supplementary Appendix S1. Full search strategy

Reported in accordance with PRISMA-S. Sources searched: PubMed/MEDLINE, Scopus, Cochrane CENTRAL, Web of Science Core Collection, ClinicalTrials.gov and the World Health Organization International Clinical Trials Registry Platform (WHO ICTRP). Coverage: inception to 31 January 2026, updated to 30 July 2026. No language, publication-status or geographic restrictions were applied. Records were deduplicated in reference-management software using title, authors, year, journal and digital object identifier, followed by manual verification.

## Deviation from the registered protocol: Embase

The registered protocol listed Embase among the intended sources. Embase was not accessible to the study team and was not searched, and no Embase records enter the record counts reported in Figure 1. Scopus and Web of Science Core Collection were searched instead; their coverage overlaps substantially with Embase, and reference lists of all included studies and of relevant systematic reviews were hand-searched, with forward citation tracking in Web of Science and Scopus. The omission of Embase is nonetheless declared as a protocol deviation and as a limitation of the review.

## Design of the strategy

The strategy was developed a priori and structured around three concept blocks: (1) acute cholecystitis; (2) EUS-guided gallbladder drainage using lumen-apposing metal stents, including cautery-enhanced and non-cautery-enhanced platforms; and (3) percutaneous cholecystostomy or percutaneous transhepatic gallbladder drainage. Controlled vocabulary was combined with free-text keywords and spelling variants to maximize sensitivity. No study-design or language filters were applied at the search stage. Animal-only studies were not excluded at the database level but were removed during screening.

## Records retrieved per source

| **Source** | **Platform** | **Date run** | **Date re-run** | **Records** |
| --- | --- | --- | --- | --- |
| PubMed/MEDLINE | NCBI | 31 January 2026 | 30 July 2026 | 66 |
| Scopus | Elsevier | 31 January 2026 | 30 July 2026 | 52 |
| Web of Science Core Collection | Clarivate | 31 January 2026 | 30 July 2026 | 42 |
| ClinicalTrials.gov | NIH | 31 January 2026 | 30 July 2026 | 17 |
| WHO ICTRP | WHO | 31 January 2026 | 30 July 2026 | 16 |
| Cochrane CENTRAL | Wiley | 31 January 2026 | 30 July 2026 | 1 |
| Embase | Not searched — protocol deviation, see above | — | — | 0 |
| Total |  |  |  | 194 |

## Search strings as run

**PubMed/MEDLINE** (run 31 January 2026; re-run 30 July 2026)

("Cholecystitis, Acute"[MeSH] OR "Cholecystitis"[MeSH] OR "acute cholecystitis"[tiab] OR "acalculous cholecystitis"[tiab]) AND ("Endosonography"[MeSH] OR "Ultrasonography, Interventional"[MeSH] OR "endoscopic ultrasound"[tiab] OR "EUS-guided"[tiab] OR "EUS-GBD"[tiab] OR "lumen-apposing metal stent*"[tiab] OR "lumen apposing metal stent*"[tiab] OR LAMS[tiab] OR AXIOS[tiab] OR "cautery-enhanced"[tiab]) AND ("Cholecystostomy"[MeSH] OR "cholecystostomy"[tiab] OR "percutaneous cholecystostomy"[tiab] OR "percutaneous transhepatic gallbladder drainage"[tiab] OR PTGBD[tiab] OR "PT-GBD"[tiab] OR "percutaneous gallbladder drainage"[tiab])

**Scopus** (run 31 January 2026; re-run 30 July 2026)

TITLE-ABS-KEY(("acute cholecystitis" OR "acalculous cholecystitis" OR cholecystitis) AND ("endoscopic ultrasound" OR endosonography OR "EUS-guided" OR "EUS-GBD" OR "lumen-apposing metal stent" OR "lumen apposing metal stent" OR LAMS OR AXIOS) AND (cholecystostomy OR "percutaneous transhepatic gallbladder drainage" OR PTGBD OR "percutaneous gallbladder drainage"))

**Web of Science Core Collection** (run 31 January 2026; re-run 30 July 2026)

TS=(("acute cholecystitis" OR "acalculous cholecystitis" OR cholecystitis) AND ("endoscopic ultrasound" OR endosonography OR "EUS-guided" OR "lumen-apposing metal stent" OR "lumen apposing metal stent" OR LAMS OR AXIOS) AND (cholecystostomy OR "percutaneous transhepatic gallbladder drainage" OR PTGBD OR "percutaneous gallbladder drainage"))

**Cochrane CENTRAL** (run 31 January 2026; re-run 30 July 2026)

```
#1 MeSH descriptor: [Cholecystitis, Acute] explode all trees
#2 ("acute cholecystitis" OR "acalculous cholecystitis"):ti,ab,kw
#3 #1 OR #2
#4 MeSH descriptor: [Endosonography] explode all trees
#5 ("endoscopic ultrasound" OR "EUS-guided" OR "lumen-apposing metal stent" OR LAMS OR AXIOS):ti,ab,kw
#6 #4 OR #5
#7 MeSH descriptor: [Cholecystostomy] explode all trees
#8 (cholecystostomy OR PTGBD OR "percutaneous gallbladder drainage"):ti,ab,kw
#9 #7 OR #8
#10 #3 AND #6 AND #9
```

**ClinicalTrials.gov** (run 31 January 2026; re-run 30 July 2026)

Condition or disease: "acute cholecystitis"
Other terms: "endoscopic ultrasound" OR LAMS OR "lumen-apposing metal stent" OR "percutaneous cholecystostomy"

**WHO ICTRP** (run 31 January 2026; re-run 30 July 2026)

Basic search: cholecystitis AND (gallbladder drainage OR cholecystostomy OR "lumen apposing")

## Supplementary search methods

Reference lists of all included studies and of relevant systematic reviews were screened by hand, and forward citation tracking was performed in Web of Science and Scopus. Where outcome data were incomplete or ambiguously reported, we recorded the ambiguity explicitly rather than imputing a value; the resulting judgements are set out study by study in Supplementary Table S5.

# Supplementary Table S0. Completed PRISMA 2020 checklist, with the section in which each item is reported.

| **#** | **PRISMA 2020 item** | **Reported in** |
| --- | --- | --- |
| 1 | Title identifies the report as a systematic review | Title |
| 2 | Structured abstract | Abstract |
| 3 | Rationale | Introduction, paragraphs 1–5 |
| 4 | Objectives with an explicit statement of the question | Introduction, final paragraph |
| 5 | Eligibility criteria | Methods, Eligibility criteria |
| 6 | Information sources with the date each was last searched | Methods, Information sources and search strategy; Supplementary Appendix S1 (per-source table with the date run and re-run) |
| 7 | Full search strategy for every source | Supplementary Appendix S1, Search strings as run |
| 8 | Selection process | Methods, Study selection and data extraction |
| 9 | Data collection process | Methods, Study selection and data extraction |
| 10a | Outcome data items and how they were sought | Methods, Outcomes; Supplementary Table S5 |
| 10b | Other variables sought | Methods, Study selection and data extraction; Table 1 |
| 11 | Risk-of-bias assessment | Methods, Risk of bias assessment; Figure 2 |
| 12 | Effect measures | Methods, Statistical analysis; Table 3 (direction column) |
| 13a | Processes for deciding eligibility for synthesis | Methods, Outcomes; Results, Ascertainment of the primary outcome |
| 13b | Data preparation and conversions | Methods, Statistical analysis |
| 13c | Tabulation and visual display | Tables 1–4; Figures 1–3E |
| 13d | Synthesis methods, model, heterogeneity and software | Methods, Statistical analysis |
| 13e | Exploration of heterogeneity | Supplementary Table S4; Supplementary Figures S2–S5 |
| 13f | Sensitivity analyses | Supplementary Table S4; Supplementary Figure S1 |
| 14 | Reporting-bias assessment | Methods, Statistical analysis (not formally testable, k < 10); Supplementary Figures S6–S7 |
| 15 | Certainty assessment | Methods, Certainty of evidence; Table 4 |
| 16a | Study selection flow | Figure 1; Results, Study selection |
| 16b | Studies excluded with reasons | Figure 1 (reasons for the 130 screening exclusions) |
| 17 | Characteristics of included studies | Table 1; Supplementary Tables S1–S3 |
| 18 | Risk of bias in the included studies | Figure 2 |
| 19 | Results of individual studies | Figures 3A–3E; Supplementary Data S1 |
| 20a | Results of syntheses: characteristics and risk of bias | Results, Study characteristics and risk of bias |
| 20b | Results of syntheses: summary estimates and precision | Table 3; Figures 3A–3E |
| 20c | Investigations of heterogeneity | Supplementary Table S4 |
| 20d | Sensitivity analyses | Supplementary Table S4; Supplementary Figure S1 |
| 21 | Reporting biases | Supplementary Figures S6–S7; Discussion, limitations |
| 22 | Certainty of evidence | Table 4; Results, Certainty of evidence |
| 23a | Interpretation in the context of other evidence | Discussion, paragraphs 1–4 |
| 23b | Limitations of the evidence | Discussion, limitations paragraph |
| 23c | Limitations of the review processes | Methods, protocol deviations; Discussion, limitations paragraph |
| 23d | Implications for practice and research | Discussion, final paragraph; Conclusion |
| 24a | Registration and registration number | Methods, Protocol registration and deviations (OSF, DOI 10.17605/OSF.IO/JW9CG) |
| 24b | Where the protocol can be accessed | Methods, Protocol registration and deviations |
| 24c | Amendments to the registered information | Methods, Protocol registration and deviations (all deviations declared) |
| 25 | Support and role of funders | Declarations, Funding |
| 26 | Competing interests | Declarations, Conflicts of interest |
| 27 | Availability of data, code and other materials | Declarations, Data availability; Supplementary Data S1 and Supplementary Code S1 |

*Completed in accordance with Page MJ et al., BMJ 2021;372:n71.*

*Item 6 is also covered by the PRISMA-S extension (Rethlefsen ML et al., Syst Rev 2021;10:39): Supplementary Appendix S1 reports the database, platform, date run, date re-run, complete search string and number of records for every source, plus the supplementary search methods (reference-list screening and forward citation tracking).*

# Supplementary Table S1. Baseline demographic and clinical characteristics.

| **Variable** | **Teoh 2020 (DRAC-1)** | **Teoh 2017** | **Irani 2017** | **Siddiqui 2019** | **Pawa 2025** | **Kesar 2026** |
| --- | --- | --- | --- | --- | --- | --- |
| Age (EUS-GBD vs PTGBD) | Mean 81.9 (SD 9.6) vs 79.8 (SD 9.6) y; P = .21 | Mean 82.7 (SD 7.9) vs 81.2 (SD 10.4) y; P = .71 | Median 65 (range 25-87) vs 75 (range 34-94) y; P = .06 | Mean 72.4 (SD 13.4) vs 60.4 (SD 17.3) y; P < .001 | Mean 73.0 (SD 14.1) vs 72.4 (SD 12.3) y; SMD 0.05 | Not reported in the available record |
| Male (EUS-GBD vs PTGBD) | 56.4% (22/39) vs 50.0% (20/40) | 50.8% (30/59) in both arms (matched on sex) | 64.4% (29/45) vs 60.0% (27/45) | 56.6% (58/102) vs 61.2% (89/146) | 60% (34/57) vs 56% (32/57) | Not reported in the available record |
| High-risk criterion | ASA >= III with age-adjusted Charlson > 5 and/or Karnofsky < 50 | Tokyo 2013, ASA >= III (83.1% in both arms), unfit for surgery | ASA IV-V (29/45 vs 36/45) or widespread malignancy | ASA IV or V; not fit for cholecystectomy | Non-surgical by ASA class, ECOG and Charlson comorbidity index | Cirrhosis, unfit for surgery |
| Calculous disease (EUS-GBD vs PTGBD) | 100% calculous (acute calculous cholecystitis was an inclusion criterion) | 100% calculous (the study was restricted to acute calculous cholecystitis) | 60.0% (27/45) vs 75.6% (34/45); acalculous 40.0% vs 24.4% | 73.7% (75/102) vs 68.0% (99/146) | 70% (40/57) in both arms; acalculous 0% vs 14% (8/57) | 63% calculous / 37% acalculous, reported for the cohort as a whole |
| Malignancy (EUS-GBD vs PTGBD) | 0% (the one randomised patient later diagnosed with gallbladder cancer was excluded from the analysis) | Not reported | 35.6% (16/45) vs 20.0% (9/45); 27.8% (25/90) overall | 18.4% (19/102) vs 15.0% (22/146) | Malignant cholecystitis 30% (17/57) vs 16% (9/57) | Not reported |
| Tokyo Guidelines grade | Grade II 34 vs 34, grade III 5 vs 6; grade I not eligible, so grade II-III = 100% | Not reported | Grade II-III 86.7% (39/45) vs 82.2% (37/45) | Not reported | Grade I-II 91% (52/57) vs 88% (50/57); P = .76 (grade III is the remainder) | Not reported |

*Values are given as EUS-GBD versus PTGBD wherever the source article reports them by arm, and for the cohort as a whole otherwise. Percentages are those printed in the source article, or are computed from the printed counts where the article prints counts only.*

*In the three-arm study, every baseline variable is reported separately for each of the three arms; the two arms analysed here are shown and the endoscopic transpapillary arm is omitted.*

*The full text of Kesar 2026 was not accessible; all of its data come from the indexed abstract (PMID 42298032), which reports the aetiology split and follow-up but not age or sex.*

# Supplementary Table S2. Pre-procedural severity and laboratory profile.

| **Variable** | **Teoh 2020 (DRAC-1)** | **Teoh 2017** | **Irani 2017** | **Siddiqui 2019** | **Pawa 2025** | **Kesar 2026** |
| --- | --- | --- | --- | --- | --- | --- |
| White cell count (×10⁹/L) | 16.7 (SD 6.8) | NR | NR | NR | NR | NR |
| Serum albumin (g/dL) | NR | NR | NR | NR | 3.02 | NR |
| Gallbladder wall thickness (mm) | NR | NR | 4 | NR | NR | NR |
| Gallbladder size (cm) | NR | 8.4 (SD 2.2) | NR | NR | NR | NR |
| Largest stone (mm) | NR | 14.2 (SD 9.4) | NR | NR | NR | NR |
| Tokyo grade distribution | Grade II 34 vs 34, grade III 5 vs 6 (grade I not eligible, so grade II–III = 100%) | NR | Grade II–III 86.7% (39/45) vs 82.2% (37/45) | NR | Grade I–II 91% (52/57) vs 88% (50/57); P = .76, grade III is the remainder | NR |
| Symptom duration (days) | 3 (IQR 2–4) | NR | 4 (range) | NR | NR | NR |

*IQR, interquartile range; NR, not reported; SD, standard deviation.*

*Values are EUS-GBD versus PTGBD wherever the source reports them by arm.*

*Note that Pawa 2025 reports the proportion with Tokyo grade I or II (mild or moderate) disease, whereas Teoh 2020 and Irani 2017 report grade II–III; the categories are not interchangeable and are therefore printed as the source states them.*

*Kesar 2026 enrolled a cirrhosis-specific cohort (63% calculous, 37% acalculous); the full text was not accessible and pre-procedural laboratory and severity variables are not reported in the indexed abstract.*

# Supplementary Table S3. EUS-GBD technical protocol and device configuration across the included studies.

| **Technical variable** | **Usual approach** | **Study-specific detail** |
| --- | --- | --- |
| Imaging guidance | EUS with or without fluoroscopy | Dual-modality guidance commonly reported |
| LAMS platform | Electrocautery-enhanced LAMS (for example Hot AXIOS) | Reported by Teoh 2020, Irani 2017 and Siddiqui 2019; Pawa 2025 used a LAMS-based strategy; Kesar 2026 reports transmural LAMS without platform detail |
| Access route | Transduodenal or transgastric | Irani 2017 predominantly transduodenal; Siddiqui 2019 mixed gastric and duodenal; Teoh 2020 both routes; Kesar 2026 not specified |
| Coaxial double-pigtail stent | Optional | Systematic in Pawa 2025; selective in Irani 2017; not stated elsewhere |
| Anaesthesia | Deep sedation or general anaesthesia | General anaesthesia commonly reported in the comparative cohorts |
| Scheduled follow-up procedures | Variable and protocol-driven | Teoh 2017 scheduled cholecystoscopy at 12 weeks in the EUS-GBD arm and tube cholecystography at 6–8 weeks in the PTGBD arm; Pawa 2025 scheduled LAMS removal at 4 weeks. These are planned procedures and are excluded from any reintervention count |

*EUS, endoscopic ultrasound; LAMS, lumen-apposing metal stent.*

*Device-level detail was not uniformly reported; entries reflect the most explicit description available in each article.*

# Supplementary Table S4. Sensitivity, robustness and post hoc exploratory analyses.

| **Analysis** | **Result** | **Interpretation** |
| --- | --- | --- |
| Primary outcome — leave-one-out: omit Teoh 2020 (DRAC-1) | k = 2; OR 0.071; 95% CI 1.26e-06–3.97e+03; I² = 78% | Not interpretable: with only two remaining studies the Hartung–Knapp interval has one degree of freedom and is effectively unbounded. The point estimate remains in the same direction |
| Primary outcome — leave-one-out: omit Teoh 2017 | k = 2; OR 0.17 (95% CI 0.001–23.76); I² = 0% | Non-significant |
| Primary outcome — leave-one-out: omit Siddiqui 2019 | k = 2; OR 0.074; 95% CI 7.04e-07–7.83e+03; I² = 80% | Not interpretable: with only two remaining studies the Hartung–Knapp interval has one degree of freedom and is effectively unbounded. The point estimate remains in the same direction |
| Primary outcome — alternative estimators | REML with Wald interval 0.098 (0.031–0.31); DerSimonian–Laird 0.098 (0.032–0.30); Mantel–Haenszel 0.10 (0.054–0.19); Peto 0.15 (0.093–0.23) | The point estimate is stable across estimators; only the pre-specified Hartung–Knapp interval includes unity. Reported to show stability of the estimate, not to supersede the pre-specified analysis. Peto is biased when effects are large and arms unbalanced and is shown for completeness only |
| Primary outcome — post hoc sensitivity adding the cirrhosis cohort's broader long-term device-burden composite | k = 4; OR 0.11 (95% CI 0.025–0.46); I² = 54% | Post hoc and exploratory. Directionally identical and statistically significant, but the added study measures long-term biliary and device-related adverse events rather than readmission, so this is a different construct |
| Technical success — ad hoc modification of the Hartung–Knapp interval | Unmodified Hartung–Knapp 0.31 (0.21–0.45), P = .001; reported (variance form of the ad hoc modification, standard error truncated at the Wald standard error, t with k − 1 df retained) 0.31 (0.087–1.11), P = .065; interval-width form of the same modification 0.31 (0.12–0.82) | Active because the estimated between-study variance was zero, which makes the unmodified Hartung–Knapp interval narrower than the Wald interval and therefore anticonservative. We report the variance form, which is the more conservative of the two and the form implemented as adhoc.hakn.ci = "se" in the analysis code. The statistical significance of this outcome depends on which form is used, and we therefore do not claim a difference |
| Technical success — estimators requiring no continuity correction | Mantel–Haenszel 0.23 (0.074–0.72); Peto 0.26 (0.098–0.68) | Concordant with the primary estimator; four of six studies contributed a zero cell |
| Adverse events within 30 days — ascertainment caveat | Irani 2017 defines its adverse-event outcome as events occurring within 30 days, but at least 4 of its 8 EUS-GBD events (one bleed at 6 months and three episodes of cholecystitis at 6, 8 and 12 months) and at least 3 of its 14 PTGBD events (cholecystitis at 2, 4 and 6 months) are dated beyond 30 days in the same article; the remaining events are undated | The tabulated counts therefore do not respect the 30-day window that the authors state, and a strictly 30-day count cannot be reconstructed from the publication. The study is retained because 30 days is the window its authors specify, but this is a further reason not to over-interpret this outcome. Restricting the analysis to the two studies whose events are time-stamped within 30 days would leave k = 2, for which a Hartung-Knapp interval is not interpretable |
| Adverse events within 30 days — source of heterogeneity | I² = 84%; the direction of effect reverses (Teoh 2020 OR 0.16; Teoh 2017 OR 1.98) | Teoh 2017 recorded three intraprocedural events, all within that institution's first 20 EUS-GBD procedures, indicating a learning-curve effect rather than a property of the technique. Influence diagnostics identify no single dominant study |
| Coaxial double-pigtail stent — meta-regression (POST HOC, EXPLORATORY, NOT PRE-SPECIFIED) | k = 6; β = 1.69 in log-odds units (ratio of odds ratios 5.44, 95% CI 1.88–15.74; P = .011) | NOT INTERPRETABLE. With fewer than ten studies and a single covariate the analysis is underpowered and at high risk of overfitting, and coaxial-stent use is confounded with study, centre and era. Reported solely for transparency in response to a reviewer request; it informs no conclusion |
| Length of hospital stay (exploratory) | Numerically shorter with EUS-GBD where reported (for example median 3 vs 4 days in Pawa 2025, P = .59) | Descriptive only; not pooled |
| Pain (exploratory) | Lower cumulative paracetamol requirement with EUS-GBD in the randomized trial (3345 vs 5165 mg, P = .034) | Descriptive only; not pooled |

*CI, confidence interval; OR, odds ratio; REML, restricted maximum likelihood.*

*All analyses in this table other than the pre-specified leave-one-out and alternative-estimator checks are exploratory. The coaxial meta-regression and the sensitivity analysis adding the cirrhosis cohort were performed post hoc and were not pre-specified in the registered protocol.*

# Supplementary Table S5. Study-level ascertainment of the primary outcome (device- or drainage-related unplanned readmission).

| **Study** | **Verbatim outcome definition** | **Ascertainment window** | **Components captured** | **EUS-GBD events / total** | **PTGBD events / total** | **Unit of counting** | **Could a patient be counted twice?** | **Source** |
| --- | --- | --- | --- | --- | --- | --- | --- | --- |
| Teoh 2020 (DRAC-1) | "The number of unplanned admissions due to gallstone related disease or the study interventions were recorded up to 1 year." | 0–12 months (all patients followed to 1 year or death; 0 lost to follow-up) | Unplanned admission only (reinterventions after 30 days tabulated separately) | 6/39 (15.4%) | 20/40 (50.0%) | Patients (printed percentages 6/39 = 15.4%, 20/40 = 50% are computed on patient denominators) | No. The endpoint has a single component, so no patient can contribute more than one event. The trial does not state explicitly how repeat admission episodes were handled; the unit is inferred from the fact that both printed percentages are computed on the patient denominators 39 and 40. | Table 2, p. 1088; abstract; Results p. 1088 |
| Teoh 2017 | "Unplanned admissions related to the intervention" | Entire follow-up (mean 451 days EUS-GBD vs 834 days PTGBD) | Unplanned admission; reinterventions are reported only as events occurring during those admissions and are not separable | 4/59 (6.8%) | 42/59 (71.2%) | Patients (4/59 = 6.8%, 42/59 = 71.2%; the paper separately notes that 16 patients had more than one admission, explicitly distinguishing patients from episodes) | No. The endpoint has a single component. The article states that 16 patients had more than one unplanned admission, which establishes that the tabulated figure counts patients and not episodes. | Table 2, p. 134; abstract p. 130; Table 4 p. 135 (reasons, sums to 42) |
| Siddiqui 2019 | "Unplanned hospital admissions"; the paper states these were admissions "due to cholecystostomy tube that required reintervention" | Median follow-up 4 months (IQR 3–9) EUS-GBD vs 3 months (IQR 3–4) PTGBD; minimum 3 months required for inclusion | Unplanned admission, with reintervention subsumed within it | 4/102 (3.9%) | 29/146 (19.9%) | Patients ("All these patients had one or more unplanned admissions"; percentages computed on 102 and 146) | No. The endpoint has a single component. The article states that "all these patients had one or more unplanned admissions", which establishes that the tabulated figure counts patients and not episodes. | Table 5, p. 1267; Results p. 1266 |
| Irani 2017 | Not reported — study does not contribute to the primary analysis | — | — | Not applicable | Not applicable | — | — | Table 4, p. 742 |
| Pawa 2025 | Not reported — study does not contribute to the primary analysis | — | — | Not applicable | Not applicable | — | — | Table 3, p. 6 |
| Kesar 2026 | Not reported — study does not contribute to the primary analysis | — | — | Not applicable | Not applicable | — | — | Abstract; PMID 42298032 |

*This table responds in full to the request for study-level detail on the primary endpoint: (i) the precise outcome definition, (ii) the ascertainment period, (iii) whether readmission, reintervention or both were captured, (iv) the event count in each arm, (v) confirmation that the unit of counting is patients and not procedures, and (vi) confirmation that no patient is counted twice.*

*On point (vi): the primary endpoint as analysed has a single component (unplanned readmission), so double counting across components cannot arise. A composite of readmission OR reintervention was considered and rejected precisely because the patient-level union of the two components cannot be computed in any included study: the randomized trial reports the two as separate rows with different ascertainment windows (admissions over 0–12 months; reinterventions only after 30 days), never cross-tabulates them, and defines reintervention so that it subsumes readmission, so only bounds are derivable (EUS-GBD 6–7 of 39; PTGBD 20–32 of 40).*

*Reasons for non-contribution. Irani 2017: Unplanned readmission is not reported for the EUS-GBD arm. The only readmission figure is 3 PTGBD patients admitted for pain from tube occlusion. Reinterventions are reported as a count of PROCEDURES (11 vs 112; 112 exceeds the 45 patients in the arm), with a per-patient mean of 0.2 ± 0.4 vs 2.5 ± 2.8; the number of patients with at least one reintervention is neither printed nor derivable. Pawa 2025: The words "readmission", "readmit", "unplanned" and "unscheduled" do not appear anywhere in the article. The nearest quantity is the total number of gallbladder interventions per patient, median 2 (IQR 2–2) vs 3 (IQR 1–6), p < 0.0001 — a count of procedures that also includes the protocol-mandated LAMS-removal endoscopy at 4 weeks. Kesar 2026: Unplanned readmission is not reported. The study reports a long-term composite of biliary tract-related and catheter/device-related adverse events (2/19 [10.5%] vs 18/43 [41.9%], p = 0.013), which is an adverse-event construct rather than a readmission construct and is therefore analysed only as a post hoc sensitivity analysis.*

# Supplementary Figures

*Supplementary Figure S1. Post hoc sensitivity analysis of the primary outcome, adding the cirrhosis cohort's broader long-term device-burden composite (k = 4). Pooled OR 0.11, 95% CI 0.025–0.46; I² = 54%. Exploratory; the added study measures long-term biliary and device-related adverse events rather than readmission.*

*Supplementary Figure S2. Baujat plot for the primary outcome. The horizontal axis shows each study's contribution to overall heterogeneity and the vertical axis its influence on the pooled estimate.*

*Supplementary Figure S3. Baujat plot for adverse events within 30 days.*

*Supplementary Figure S4. Cook's distances for the primary outcome.*

*Supplementary Figure S5. Cook's distances for adverse events within 30 days. No single study meets a threshold for disproportionate leverage.*

*Supplementary Figure S6. Funnel plot for the primary outcome. Exploratory only: no formal test of asymmetry was performed because fewer than ten studies contributed.*

*Supplementary Figure S7. Funnel plot for adverse events within 30 days. Exploratory only; fewer than ten studies contributed.*
